# CRISPR/Cas9-mediated efficient targeted mutagenesis in Chardonnay (*Vitis vinifera* L.)

**DOI:** 10.1038/srep32289

**Published:** 2016-08-31

**Authors:** Chong Ren, Xianju Liu, Zhan Zhang, Yi Wang, Wei Duan, Shaohua Li, Zhenchang Liang

**Affiliations:** 1 Beijing Key Laboratory of Grape Science and Enology and Key Laboratory of Plant Resource, Institute of Botany, the Chinese Academy of Sciences, Beijing 100093, PR China; 2 University of Chinese Academy of Sciences, Beijing 100049, PR China; 3 Sino-Africa Joint Research Center, Chinese Academy of Sciences, Wuhan 430074, PR China

## Abstract

The type II clustered regularly interspaced short palindromic repeats/CRISPR-associated protein 9 system (CRISPR/Cas9) has been successfully applied to edit target genes in multiple plant species. However, it remains unknown whether this system can be used for genome editing in grape. In this study, we described genome editing and targeted gene mutation in ‘Chardonnay’ suspension cells and plants via the CRISPR/Cas9 system. Two single guide RNAs (sgRNAs) were designed to target distinct sites of the L-idonate dehydrogenase gene (*IdnDH*). CEL I endonuclease assay and sequencing results revealed the expected indel mutations at the target site, and a mutation frequency of 100% was observed in the transgenic cell mass (CM) as well as corresponding regenerated plants with expression of sgRNA1/Cas9. The majority of the detected mutations in transgenic CM were 1-bp insertions, followed by 1- to 3-nucleotide deletions. Off-target activities were also evaluated by sequencing the potential off-target sites, and no obvious off-target events were detected. Our results demonstrated that the CRISPR/Cas9 system is an efficient and specific tool for precise genome editing in grape.

Targeted genome editing (TGE) using site-specific nucleases (SSNs) promises to be critical to basic and applied biology research. These powerful SSN tools, comprising zinc-finger nucleases (ZFNs), transcription activator-like effector nucleases (TALENs), and clustered regulatory interspaced short palindromic repeats (CRISPR)/CRISPR-associated protein 9 (Cas9) system (CRISPR/Cas9), introduce targeted DNA double-strand breaks (DSBs) and subsequently trigger DNA repair pathways involving either non-homologous end-joining (NHEJ) or homologous recombination (HR)^1^. Both ZFNs and TALENs, which consist of customizable ZF/TALE DNA-binding domains (DBDs) with *Fok*I cleavage domains, have been proven successful in targeted genome modification^2^,^3^,^4^. However, disadvantages such as the time required for designing the constructs make these two methods difficult and expensive to use^5^,^6^.

Recently, the RNA-guided CRISPR/Cas9 system has been widely used in TGE. There are three types of CRISPR/Cas9 systems (I, II and III), and the type II system with a single Cas9 protein has emerged as a simple but highly efficient tool for TGE^7^,^8^. The Cas9 nuclease is guided by a duplex formed by CRISPR RNA (crRNA) and trans-activating crRNA (tracrRNA) which cleaves the complementary sequence of the crRNA accompanied by an NGG protospacer adjacent motif (PAM)^9^,^10^. The PAM is indispensable for target recognition as it helps the crRNA-tracrRNA-Cas9 complex combine with the target sequence^11^,^12^. In an engineered CRISPR/Cas9 system, the crRNA-tracrRNA duplex has been fused into an artificial single guide RNA (sgRNA) containing a 20-nucleotide (nt) sequence that determines the DNA target site^7^,^10^,^13^. To date, the CRISPR/Cas9 system has been adopted for TGE successfully in many organisms, including mammalian cells^10^,^13^, yeast^14^, zebrafish embryos^15^, mice^16^, and numerous herbaceous plants such as *Arabidopsis*^17^,^18^,^19^, tobacco^19^,^20^, rice^17^,^21^,^22^, wheat^23^, maize^6^, sorghum^23^, soybean^24^, and tomato^25^,^26^. This genome modification is heritable, and the sequence changes in the first generation of stable herbaceous plant transformants persist into the next generation^27^,^28^. In addition, the transient expression of Cas9 and sgRNA in rice and wheat protoplasts also successfully directs genome modification^29^. Nevertheless, the CRISPR/Cas9 system has rarely been reported to effectively work in perennial plants except for poplar and citrus^30^,^31^.

As one of the most important fruit crops worldwide, grape has tremendous economic value. Since the whole-genome sequence of grape (*Vitis vinifera* L.) was released in 2007^32^, tremendous gene resources were available for functional studies in *Vitis*. Therefore, a useful and efficient system is crucial for gene functional studies as well as genetic engineering in grape. Ectopic manipulation of gene expression either via overexpression or knock-out is a very useful strategy for gene functional studies^33^. Random insertional mutagenesis that proved useful in model plants remains difficult for grape due to its biological characteristics^34^. As a rapid and efficient tool to generate targeted mutations, the CRISPR/Cas9 system is becoming the primary tool for gene editing in plants. However, there is no report on the efficacy of CRISPR/Cas9 in grape to date, so it is necessary to determine if it can be a useful tool in *Vitis*.

Here we report on targeted gene editing in grape via the CRISPR/Cas9 system by introduction of a single plasmid with a specific sgRNA into grape cells. Though *Agrobacterium*-mediated transformation of grapevine has been reported previously^35^, the efficiency and reliability of this technology is presently low, and most grape cultivars are recalcitrant for plant regeneration^34^,^36^,^37^. Additionally, somatic mutations frequently occur during the tissue culture-based transformation and many abnormal plantlets may be generated from somatic embryos^38^,^39^. Therefore, it is difficult and time-consuming to obtain enough grapevines for our studies, so ‘Chardonnay’ suspension cells were adopted as our main experimental material, and the L-idonate dehydrogenase gene (*IdnDH*, LOC100232980), which controls the biosynthesis of tartaric acid (TA) in grape^40^, was chosen as the target gene to be disrupted site-specifically in suspension cells of ‘Chardonnay’. Knocking out or silencing the gene resulted in failure of TA biosynthesis or accumulation in suspension cells. Mutagenesis in a transgenic cell mass (CM) and resulting grape plants was detected by CEL I endonuclease assays and Sanger sequencing, respectively. The content of TA in the transgenic CM was also measured to determine the effect of mutagenesis. The expression levels of *Cas9* and sgRNAs were measured by quantitative real-time PCR (qRT-PCR). Furthermore, off-target events were evaluated. Taken together, our data demonstrated that the CRISPR/Cas9 system could efficiently induce targeted mutations in ‘Chardonnay’ suspension cells as well as its regenerated plants, indicating the feasibility of this system as a powerful tool for genome modification in grape.

## Results

### sgRNA design and CRISPR/Cas9 expression vector construction

**Figure 1:**
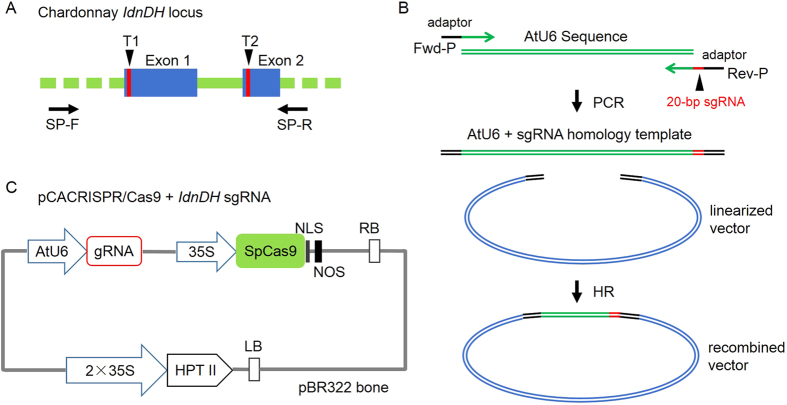
Selection of target sites in the *IdnDH* gene and construction of Cas9/sgRNA expression vector. (**A**) Schematic illustrating the target sites in the *IdnDH* coding sequence. Blue boxes indicate exons and green lines denote introns. T1 and T2 are selected target sites. SP-F and SP-R are primers used for PCR amplification. (**B**) Construction process of Cas9/sgRNA expression vector. (**C**) Schematic diagram of the assembled Cas9/sgRNAs expression vector for stable transformation. The vector used in this study was named pCACRISPR/Cas9.

The L-idonate dehydrogenase gene (*IdnDH*) was selected as the target of Cas9 nuclease. An approximately 1-kb DNA fragment of *IdnDH* was cloned and sequenced, and the result showed that the sequence of *IdnDH* in ‘Chardonnay’ was identical to that of its homologous gene in ‘Pinot Noir’ PN40024 (data not shown). Two 20-bp sequences with tandem guanosine nucleotides (PAM) on their 3’-regions in the *IdnDH* sequence were chosen as sgRNA complementary sites, including one in the first exon and the other in the second exon (Fig. 1A). The construction process of the pCACRISPR/Cas9 binary vectors is shown in Fig. 1B. In brief, the *Arabidopsis* U6 promoter (AtU6) and sgRNA were first combined by PCR, and then the sgRNA expression cassette with adaptors was inserted into the linearized vector by the HR method. Finally, two binary vectors were constructed, where the expression of Cas9 and sgRNA was driven by the CaMV 35S promoter and AtU6, respectively (Fig. 1C).

### Screening and identification of positive transgenic CM and plants

**Figure 2:**
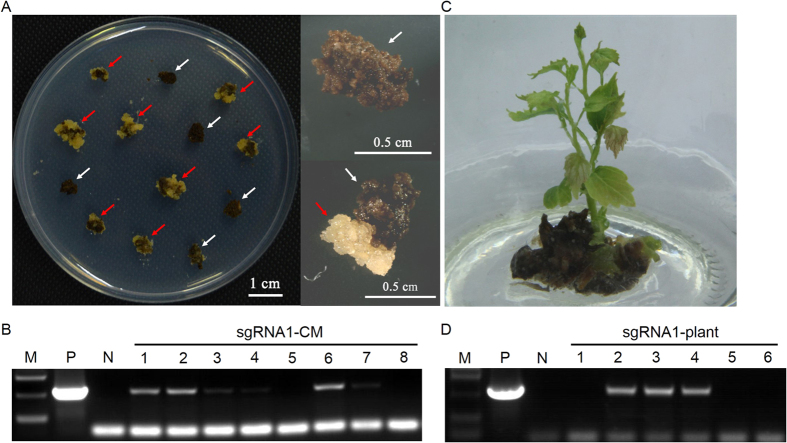
Screening and identification of positive transgenic cell mass (CM) and plantlets. (**A**) Yellowish resistant CMs generated on selective medium. (**B**) Identification of exogenous T-DNA insertion in sgRNA1-CMs. Genomic DNA of sgRNA1-CMs was extracted and used as templates for PCR with specific primers for the hygromycin resistant gene. The plasmid of the constructed vector and wild-type DNA were used as a positive control (P) and a negative control (N), respectively. Lanes 1–8 represent different sgRNA1-CMs. (**C**) Grape plantlets regenerated from transgenic CMs. (**D**) Identification of exogenous T-DNA insertion in sgRNA1-plants. Genomic DNA was extracted from plants and used as templates for PCR with specific primers for the hygromycin resistant gene. The plasmid of the constructed vector and wild-type DNA were used as a positive control (P) and a negative control (N), respectively. Lanes 1–6 represent different individual sgRNA1-plants.

After *A. tumefaciens*-mediated transformation and screening on selective medium, yellowish resistant CMs were generated, whereas non-resistant CMs turned brown and were unable to proliferate (Fig. 2A). A total of 62, 58 and 15 independent resistant CMs in empty vector (without 20-bp sgRNA sequence, EV-*IdnDH*), sgRNA1-*IdnDH* and sgRNA2-*IdnDH* vectors, respectively (Table 1). Identification of these CMs by PCR using specific primers for the hygromycin resistant gene (Supplementary Table S2) revealed that 27, 21, and 3 of the examined CMs contained exogenous T-DNA insertions, respectively (Fig. 2B, Table 1). The transgenic CMs identified were subsequently transferred into liquid CSM medium with 10 mg/L hygromycin for rapid propagation. Some of the cells were transferred onto regeneration medium to generate transgenic plants. Finally, 4, 6 and 0 independent normal shoots were generated in EV-*IdnDH*, sgRNA1-*IdnDH* and sgRNA2-*IdnDH*, respectively (Fig. 2C, Table 1). Among these shoots, 25% (1 out of 4) of the EV-*IdnDH* plantlets (EV-plant) and 50% (3 out of 6) of the sgRNA1-*IdnDH* plantlets (sgRNA1-plant) were identified with exogenous T-DNA insertions (Fig. 2D, Table 1).

### Detection of CRISPR/Cas9-mediated mutagenesis in the *IdnDH*

**Figure 3:**
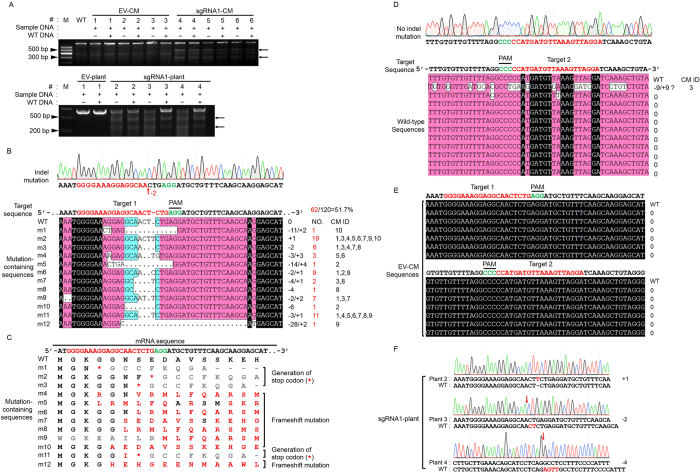
Analysis of induced mutations in target sequence. (**A**) Detection of target mutations by CEL I endonuclease assay. The target fragments were amplified by PCR from genomic DNA of wild-type (WT), EV-*IdnDH* and sgRNA1-*IdnDH*, and then digested with CEL I endonuclease. Arrows indicate the digested fragments by CEL I. +: PCR products were added. −: no PCR products were added. Lanes 1–6 represent different samples. (**B**) An example chromatogram showing a microdeletion, as well as representative sequences with indel mutations identified from 120 clonal amplicons of sgRNA1-CMs. The homologous nucleotides are shaded and different colors indicate different homology levels. The nucleotides with a homology level of 100% are shaded in black and those with a homology level of ≥75% and ≥50% are shaded in red and blue, respectively. Red numbers on the right indicate the number of detected clones with the same mutation type. (**C**) Mutations of amino acids in corresponding mutated sequences in (**B**). (**D**) An example chromatogram and representative sequences identified from 120 clonal amplicons of sgRNA2-CMs. (**E**) The sequencing results of 36 clonal amplicons of EV-CMs. (**F**) The chromatograms showing the indel mutations in sgRNA1-plants.

Mutations in positive CMs were first detected by using the CEL I endonuclease assay. The DNA fragments containing target sites from transgenic CMs of sgRNA1-*IdnDH* (sgRNA1-CM) were digested with CEL I enzyme and two different fragments were produced, whereas the DNA fragments from wild-type (WT) as well as EV-CMs were not digested with CEL I (Fig. 3A). Similar results were also obtained with CEL I endonuclease assays of DNA fragments from regenerated shoots (Fig. 3A). These results suggested that the DNA fragments from sgRNA1-CM and sgRNA1-plant had mutations, and those from EV-CM and EV-plant might be wild-type. Notably, the presence or absence of WT DNA made no difference in the results, indicating that these CMs may be mosaics (100%, 21/21) and the harvested plants might be heterozygotes or chimeras (100%, 3/3).

To accurately verify the results, the modification of DNA fragments was detected by sequence analysis of 10 EV-CMs, 10 sgRNA1-CMs and 3 sgRNA2-CMs, respectively. As the CM was chimeric, more than 12 clones of PCR amplicons from each sample were sequenced. The results showed that fragments from sgRNA1-CMs contained indel mutations at the target site (Fig. 3B). All the examined sgRNA1-CMs contained deletion or insertion mutations at the target site, with a high mutation rate of 100% (10/10). The high mutation rate was partially due to the fact that the CRISPR/Cas9-induced editing events mainly take place in the transformed cells^41^. Most mutations (15.83%, 19 out of 120 clones) were 1-bp (T) insertions, which was consistent with previous reports in *Arabidopsis* and rice^27^,^28^, followed by short nucleotide deletions (≤3 bp) (Fig. 3B). Only 0.83% (1/120) of the mutations exhibited >20-bp deletions. In addition, there were mutations of 11-bp and 14-bp deletions (Fig. 3B). Most indel mutations in sgRNA1-CMs led to frame shifts of the open reading frame (ORF) and the majority of encoded amino acid were altered (Fig. 3C). Intriguingly, among the 12 kinds of mutation-containing sequences, 4 (33.33%, 4/12) exhibited stop codons on the 5’-region of their mRNA sequences (Fig. 3C) which would result in premature translation termination. However, most DNA fragments from sgRNA2-CMs showed no changes at the target site (Fig. 3D), except that one fragment from sgRNA2-CM 3 contained multiple nucleotide mutations within the 20-bp target and adjoining sequence, which was different from typical mutations induced by CRISPR/Cas9^26^,^27^,^28^. As expected, no mutations were detected in DNA fragments from EV-CMs (Fig. 3E), suggesting the indispensable role of 20-bp sgRNA sequence in TGE mediated by Cas9/sgRNA. The modification of the targeted sequence in sgRNA1-plants was investigated as well. The DNA fragments from three independent plantlets (plants 2, 3 and 4), which were regenerated from sgRNA1-CM 1, sgRNA1-CM 4 and sgRNA1-CM 8, respectively, contained a 1-bp insertion, or 2-bp and 4-bp deletions at the target site, respectively (Fig. 3F). The results were consistent with that in sgRNA1-CMs (Fig. 3B).

### Measurement of TA contents in transgenic CM

The TA content of transgenic CMs was measured to evaluate the effect of mutagenesis in *IdnDH*. The TA contents in EV-CM (EC) exhibited no significant difference. Among the four sgRNA1-CMs, three exhibited significant differences in TA content compared to that of EC-1 (Table 2). Actually, indels of −3/+3 bp or −4/+1 bp may not lead to frameshifts of the ORF, consequently resulting in no defect in phenotype. More significantly, the presence of wild-type cells or cells without mutations in the mosaic CM may affect the determination of TA content. In short, the results suggested that the N’ terminus of IdnDH was an effective target site and frameshift mutations of *IdnDH* were likely to disrupt its expression.

### Expression analysis of *Cas9* and sgRNAs

qRT-PCR was conducted to investigate the expression profiles of *Cas9* and sgRNA. There was not much difference in expression levels of *Cas9* driven by the 35S promoter between EV-CMs and sgRNA1-CMs. However, the expression levels of sgRNA1 driven by the AtU6 promoter in sgRNA1-CMs were highly increased (Supplementary Figure S1). Both *Cas9* and sgRNA2 in sgRNA2-CMs exhibited lower expression abundance compared to that of EC-1, with the exception being sgRNA2-1, where the transcript level of sgRNA2 was about 4.7-fold that of EC-1 (Supplementary Figure S1). However, the three sgRNA1 plantlets had higher expression levels of *Cas9* and sgRNA1 compared to that of EV-plant (Supplementary Figure S1). These results suggested that the abundance of sgRNA as well as *Cas9* was significant in triggering genome editing.

### Off-target analysis

In two sgRNA-*IdnDH* constructs, only the sgRNA1-*IdnDH* construct successfully generated mutations at the target site (Fig. 3). To detect off-target events in transgenic CMs, potential off-target sites predicted with the online tool CRISPR-P were selected for further study (Table 3). Genomic DNA extracted from those CMs (10 sgRNA1-CMs and 3 sgRNA2-CMs) adopted for sequence analysis was used as a template to amplify DNA fragments containing the putative off-target sites by PCR with specific primers (Supplementary Table S2). At least 10 clones of PCR amplicons from each CM were sequenced. Apart from variation of some nucleotides, no mutation was found at the putative off-target sites in the tested transgenic CMs (Table 3 and Supplementary Figure S2). The results indicated that the mutagenesis induced by CRISPR/Cas9 system in grape was highly specific.

## Discussion

As a simple but efficient site-directed genome editing technology, CRISPR/Cas9 system has been widely used for genome editing in multiple organism^4^,^6^,^8^,^15^. However, the efficiency and application of this system in grape (grape cells or grapevines) has not been reported to date. Like most woody plants, grape is classically improved through vegetative methods or conventional breeding. Application of any gene technology to grape requires an efficient transformation system that can be applied to a wide range of cultivars^42^. Despite significant progress in genetic engineering based on *Agrobacterium*-mediated transformation, some cultivars of *Vitis vinifera* are still recalcitrant to transformation^37^. Furthermore, efficiency and reliability of this technology has been low, and many factors such as *Agrobacterium* strain, culture medium and grape genotype can play an important role in transformation efficiency^34^,^37^. Moreover, it takes a relatively long time (over a 32 week period) to obtain transgenic embryos from a small amount of embryogenic callus, during which somatic mutations frequently occur and abnormal plantlets are usually generated^36^,^38^,^39^,^42^. Hence, a stable and efficient transformation and regeneration method is needed for application of the CRISPR/Cas9 system in grape.

In this study, the gene *IdnDH*, which regulates the stable accumulation of TA in grape cells, was selected as the target gene to verify the efficacy of the CRISPR/Cas9 system in ‘Chardonnay’. Two different sgRNA expression cassettes were inserted into the binary pCACRISPR/Cas9 vector via HR, and were then used to transform ‘Chardonnay’ suspension cells by infection with *A. tumefaciens*. Identification of exogenous T-DNA insertion by PCR in the CMs obtained revealed an average transformation rate of 37.78% (Table 1). CEL I endonuclease assays and Sanger sequencing showed that the sgRNA1 had successfully directed the target-editing in transgenic CMs and plants (Fig. 3A,B,F), whereas the sgRNA2 did not result in indel mutation at the target site (Fig. 3D). However, there was one piece of sequence from sgRNA2-CM 3 containing several nucleotide mutations (Fig. 3D). Interestingly, most nucleotides of the 12-nucleotide ‘seed sequence’ located at the target site and adjoining the PAM were conserved, and there was no deletion occurred at the 4th base of the PAM, which have been regarded as one of the typical mutations induced by CRISPR/Cas9 system^27^,^28^. Importantly, it cannot be ruled out that these nucleotide mutations might be introduced by the DNA polymerase during PCR amplification. Actually, the greatest likelihood is that sgRNA2 was inactive for on-target editing^43^. Thus, the sgRNA2 failed to induce site-directed cleavage in grape cells. In contrast, a high mutation rate of 100% was detected in the sgRNA1-CMs tested (Fig. 3B, Table 1). In three sgRNA1 plantlets, insertion and deletion mutations were detected at the target site (Fig. 3F). Previous reports have shown that the GC content of target sequences may influence editing efficiency in plants, and a higher GC content (>50%) could generate high editing efficiency while a lower GC content (≤40%) results in low editing efficiency^26^,^38^,^44^. In our study, the GC content of sgRNA1 and sgRNA2 was 60.0% and 35.0%, respectively (Supplementary Table S1). Thus, the high GC content of sgRNA1 may contribute to the high mutation rate, and the failure of sgRNA2 to edit might be due to the low GC content. In addition, CRISPR/Cas9-induced editing efficiency in plants may also be affected by expression levels of *Cas9* and sgRNA^26^,^38^,^45^. The results of expression analyses showed that the expression level of *Cas9* increased in sgRNA1-CMs and sgRNA1-plants, but was down-regulated in sgRNA2-CMs (Supplementary Figure S1). In rice and *Arabidopsis*, transgenic plants with no targeted mutations generally have very low expression levels of sgRNA^38^. The transcript level of sgRNA1 was extremely high, and the signal was over 2000-fold that of EC-1(Supplementary Figure S1). However, the transcript abundance of sgRNA2 was lower than that of EC-1, except that sgRNA2-1 had a higher level of sgRNA but a lower expression level of *Cas9* (Supplementary Figure S1). These results suggested that the GC content and the abundance of sgRNA and *Cas9* were significant limiting factors for genome editing.

Off-target mutations are the main concern in the application of the CRISPR/Cas9 system. The probability of off-target events induced by the CRISPR/Cas9 system varies among different organisms^46^,^47^. Generally, the probability of off-target mutations is low in plants^28^,^48^,^49^. In our study, no off-target events were observed in the tested putative off-target sites by sequence assays (Supplementary Figure S2), suggesting the high specificity of the CRISPR/Cas9 system in grape. There are several factors affecting the specificity of RNA-guided genome editing. The selection of the target sequence is vital for the specificity and efficacy of the CRISPR/Cas9 system. As mentioned above, target sequences with a high GC content (50–70%) has relatively high editing efficiency^38^, yet a high GC content may potentially lead to the risk of off-targeting^44^. Furthermore, the secondary structure of sgRNAs and local chromatin structure may affect the specificity of CRISPR/Cas9 system^8^,^38^,^50^. The specificity of Cas9 also depends upon the relative abundance of effective Cas9-sgRNA complexes with respect to the effective target concentration^44^. As the PAM and ‘seed sequence’ are necessary and significant for the specific binding of Cas9, it is the most effective strategy to determine a highly specific target sequence with the help of online tools^51^. In addition, the use of Cas9 nickase, which consists of an inactivating mutation in the endonuclease cleavage domains and produces single-strand breaks, could reduce off-target mutations^7^,^52^,^53^. The newly found CRISPR/Cpf1 system can recognize the T- and T/C-dependent PAMs and produce a staggered DSB to activate the DNA repair process, through which the efficiency and specificity of the system could be improved^54^,^55^.

In this study, the CRISPR/Cas9 system was used to successfully generate TGE for *IdnDH* in ‘Chardonnay’ suspension cells and regenerated plantlets. The contents of TA, as well as the expression levels of *Cas9* and sgRNAs, were measured to verify the results of TGE. No off-target mutations were detected in the tested putative off-target sites, suggesting a specificity of the CRISPR/Cas9 system in grape. In short, our study proves that the CRISPR/Cas9 system is a promising and useful tool for investigating the function of candidate genes and conducting genetic improvement in grape.

## Methods

### Cloning of *IdnDH* gene

The primers (Supplementary Table S2) used for amplification of genomic DNA fragments of *IdnDH* in ‘Chardonnay’ were designed based on its homologous gene (LOC100232980) sequence in ‘Pinot Noir’. The PCR reaction was carried out with High-Fidelity DNA polymerase KOD-plus Neo (TOYOBO, Japan) in a total volume of 50 μL at 95 °C for 5 min; 34 cycles of 95 °C for 15 s, 60 °C for 25 s and 68 °C for 30 s; followed by a final extension at 68 °C for 10 min. The PCR product was cloned into pLB-Simple vector (TIANGEN, China) and around 5 clones were sequenced.

### Design of sgRNA and assembly of CRISPR/Cas9 construct

The verified sequence of *IdnDH* was input into the online tool Grape-CRISPR^56^ (http://biodb.sdau.edu.cn/gc/) and CRISPR-P^57^ (http://cbi.hzau.edu.cn/cgi-bin/CRISPR#) to find potential Cas9 target sites, and the output target sites were selected for designing target sgRNAs based on their location in the gene and off-target possibilities. The backbone of the pCACRISPR/Cas9 vector carrying plant-optimized *Streptococcus pyogenes* Cas9 protein coding gene was derived from the pP1C.4 vector (Genloci, China). For constructing the AtU6-sgRNA cassette, the 20-bp sgRNA sequence as well as an adaptor were synthesized as reverse primers for amplification of AtU6 promoter fragments. The amplified AtU6-sgRNA fragments containing adaptors could be inserted into the homologous sites of the linearized pCACRISPR/Cas9 binary vector via HR.

### Growth, transformation of Chardonnay suspension cells and regeneration of plants

The anthers of ‘Chardonnay’ at the mid-late uninucleate stage were placed on induction medium (MS basal medium supplemented with 2 mg/L 6-benzyladenine [6-BA], 1 mg/L 2,4-dichlorophenoxyacetic acid [2,4-D], 60 g/L sucrose, 3 g/L phytagel, pH 5.8) to induce embryogenic callus. The suspension cells, derived from induced embryogenic callus, were cultured in 250 mL flasks fitted with 50 mL of liquid CSM medium (MS basal medium supplemented with 0.5 g/L glutamic acid, 1 mg/L 2-naphthoxyacetic acid [NOA], 5.0 mL/L glycerol, 20 g/L maltose, pH 5.8), and shaken at 117 rpm at 26 ± 1 °C in the dark. All the suspension cells were sub-cultured every 7 days. The final binary vectors were introduced into the *Agrobacterium* strain EHA105 by the freeze-thaw method. For *A. tumefaciens*-mediated transformation, the cultures of suspension cells were collected by short centrifugation, and then inoculated with a prepared *A. tumefaciens* suspension. The explants were transferred onto selective medium (MS basal medium supplemented with 1 mg/L 2,4-D, 2 mg/L 6-BA, 1 mg/L NOA, 3 g/L phytagel, pH 5.8) with 10 mg/L hygromycin until resistant CMs were generated. These resistant CMs were subsequently transferred into liquid CSM medium with 10 mg/L hygromycin for rapid propagation. Some of sub-cultured cells were transferred onto regeneration medium (MS basal medium supplemented with 0.2 mg/L kinetin, 0.1 mg/L NOA, 30 g/L sucrose, 3 g/L phytagel, pH 5.8) to generate plants.

### Extraction of genomic DNA

Genomic DNA was extracted from WT and resistant CM as well as plants using the typical CTAB method as previously described^30^.

### Identification of exogenous T-DNA insertion by PCR

The specific primers for the hygromycin resistant gene (Supplementary Table S2) were used for PCR. The PCR product was separated on an ethidium bromide-stained agarose gel (1.0%) and then recovered and cloned into the pLB-Simple vector for Sanger sequencing.

### CEL I endonuclease assay and sequencing analysis

The prepared genomic DNA was used as a template to amplify genomic fragments containing the target sites. The sequences of specific primers (SP) used for PCR were identical to that of cloning primers. For CEL I assay, the PCR products were digested with the Surveyor Mutation Detection Kit (Transgenomic, USA) according to the manufacturer’s instruction. The PCR products were further verified by Sanger sequencing.

### Off-target analysis

The potential off-target sites of the target sequence were predicted simultaneously during the process of designing sgRNAs with the online tool of CRISPR-P. Two top-ranking potential off-target sites of each sgRNA were selected for off-target analysis, respectively. The genomic DNA fragments containing the potential off-target sites were amplified using specific primers (Supplementary Table 2) and then analyzed by Sanger sequencing.

### Quantitative RT-PCR analysis

Total RNA was extracted from ‘Chardonnay’ CM and regenerated plants using TRIzol reagent (Invitrogen, USA). The first cDNA was synthesized with 1 μg of RNA using the HiScript Q RT SuperMix for qPCR (+gDNA wiper) kit (Vazyme, China) according to the manufacturer’s instruction. qRT-PCR was performed in a volume of 20 μL on a CFX96 Real-Time System (Bio-Rad, USA) with *Cas9* and sgRNAs specific primers (Table S2). The grape *Actin1* (accession number AY680701) was used as internal control. The relative expression level was calculated using the 2^−ΔΔCT^ method^58^.

### Determination of TA by HPLC

To measure the content of TA, 1 g fresh weight of CM powder was homogenized with 6 mL ultrapure water and incubated for 2 h, then centrifuged at 20,000×g for 10 min. A 2 mL volume of supernatant was removed and passed through a C18 extraction column (Waters, Milford, MA) and 0.22 μm filter, and organic acids were separated by HPLC. The mobile phase was 20 mM KH_2_PO_4_ adjusted to pH 2.4 with phosphoric acid at a flow rate of 0.8 mL/min.

## Additional Information

**How to cite this article**: Ren, C. *et al*. CRISPR/Cas9-mediated efficient targeted mutagenesis in Chardonnay (*Vitis vinifera* L.). *Sci. Rep.* **6**, 32289; doi: 10.1038/srep32289 (2016).

## Supplementary Material

Supplementary Information

## Figures and Tables

**Table 1:** Number and percentage of examined transgenic CMs and plants with exogenous T-DNA and mutations.

Vector	No. of obtained CMs	No. of CMs with T-DNA	No. of examined CMs	No. of CMs with mutation	Mutation rate in CMs (%)	No. of regenerated plants	No. of plants with T-DNA	No. of plants with mutation	Mutation rate in plants (%)
AtU6-(None)-CaMV35S-Cas9	62	27	10	0	0.0	4	1	0	0.0
AtU6-sgRNA1-CaMV35S-Cas9	58	21	10	10	100.0	6	3	3	100.0
AtU6-sgRNA2-CaMV35S-Cas9	15	3	3	1	33.3	0	0	0	0.0

**Table 2:** Determination of TA in EV- and sgRNA1-CM.

Samples	CM ID	Content of TA (mg/g)	Range of values	P-value
EC-1	1	91.73 ± 3.98	87.89–97.21	ND
EC-2	3	90.35 ± 5.78	88.36–95.95	0.70
sgRNA1-1	1	83.53 ± 2.08*	81.52–85.67	0.02
sgRNA1-2	2	87.96 ± 2.97	84.60–90.25	0.23
sgRNA1-3	4	82.73 ± 1.96*	80.82–84.73	0.02
sgRNA1-4	9	58.67 ± 1.17**	57.35–59.75	0.00

The content of TA was analyzed by HPLC and the data obtained from at least three replicates are presented as mean values ± SD. A P-value was determined by Student’s *t*-test. Asterisks indicate statistically significant differences between EC-1 and sgRNA1-CM. *P < 0.05 and **P < 0.01. ND, not determined.

**Table 3:**
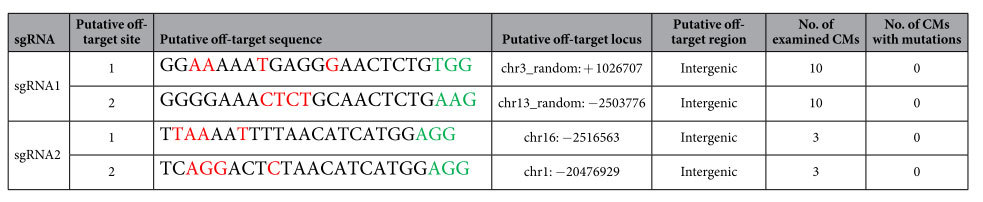
Analysis of mutation in potential off-target sites.

PAM sequence (NGG and the analogue NAG) is indicated in green. Mismatched nucleotides are marked in red.

## References

[b1] Symington L. S. & Gautier J. Double-strand break end resection and repair pathway choice. Annu. Rev. Genet. 45, 247–271 (2011). 21910633 10.1146/annurev-genet-110410-132435

[b2] Townsend J. A. . High-frequency modification of plant genes using engineered zinc-finger nucleases. Nature 459, 442–445 (2009). 19404258 10.1038/nature07845 PMC2743854

[b3] Shan Q. . Rapid and efficient gene modification in rice and Brachypodium using TALENs. Mol. Plant 6, 1365–1368 (2013). 23288864 10.1093/mp/sss162 PMC3968307

[b4] Zhang Y. . Transcription activator-like effector nucleases enable efficient plant genome engineering. Plant Physiol. 161, 20–27 (2013). 23124327 10.1104/pp.112.205179 PMC3532252

[b5] DeFrancesco L. Erratum: Move over ZFNs. Nat. Biotechnol. 30, 112 (2012).

[b6] Chen K. L. & Gao C. Targeted genome modification technologies and their applications in crop improvements. Plant Cell Rep. 33, 575–583 (2014). 24277082 10.1007/s00299-013-1539-6

[b7] Cong L. . Multiplex genome engineering using CRISPR/Cas systems. Science 339, 819–823 (2013). 23287718 10.1126/science.1231143 PMC3795411

[b8] Wu X.. Genome-wide binding of the CRISPR endonuclease Cas9 in mammalian cells. Nat. Biotechnol. 32, 670–676 (2014). 24752079 10.1038/nbt.2889 PMC4145672

[b9] Gaj T., Gersbach C. A. & Barbas C. R. ZFN, TALEN, and CRISPR/Cas-based methods for genome engineering. Trends Biotechnol. 31, 397–405 (2013). 23664777 10.1016/j.tibtech.2013.04.004 PMC3694601

[b10] Jinek M. . A programmable dual-RNA-guided DNA endonuclease in adaptive bacterial immunity. Science 337, 816–821 (2012). 22745249 10.1126/science.1225829 PMC6286148

[b11] Nishimasu H. . Crystal structure of Cas9 in complex with guide RNA and target DNA. Cell 156, 935–949 (2014). 24529477 10.1016/j.cell.2014.02.001 PMC4139937

[b12] Sternberg S. H., Redding S., Jinek M., Greene E. C. & Doudna J. A. DNA interrogation by the CRISPR RNA-guided endonuclease Cas9. Nature 507, 62–67 (2014). 24476820 10.1038/nature13011 PMC4106473

[b13] Mali P. . RNA-guided human genome engineering via Cas9. Science 339, 823–826 (2013). 23287722 10.1126/science.1232033 PMC3712628

[b14] Wei C. . TALEN or Cas9-rapid, efficient and specific choice for genome modifications. J. Genet. Genomics 40, 281–289 (2013). 23790627 10.1016/j.jgg.2013.03.013

[b15] Hwang W. Y. . Efficient genome editing in zebrafish using a CRISPR-Cas system. Nat. Biotech. 31, 227–229 (2013). 10.1038/nbt.2501 PMC3686313 23360964

[b16] Yang H. . One-step generation of mice carrying reporter and conditional alleles by CRISPR/Cas mediated genome engineering. Cell 154, 1370–1379 (2013). 23992847 10.1016/j.cell.2013.08.022 PMC3961003

[b17] Feng Z. . Efficient genome editing in plants using a CRISPR/Cas system. Cell Res. 23, 1229–1232 (2013). 23958582 10.1038/cr.2013.114 PMC3790235

[b18] Mao Y. . Application of the CRISPR-Cas system for efficient genome engineering in plants. Mol. Plant 6, 2008–2011 (2013). 23963532 10.1093/mp/sst121 PMC3916745

[b19] Li J. F. . Multiplex and homologous recombination mediated genome editing in Arabidopsis and Nicotiana benthamiana using guide RNA and Cas9. Nat. Biotechnol. 31, 688–691 (2013). 23929339 10.1038/nbt.2654 PMC4078740

[b20] Nekrasov V., Staskawicz B., Weigel D., Jones J. D. G. & Kamoun S. Targeted mutagenesis in the model plant Nicotiana benthamiana using Cas9 RNA-guided endonuclease. Nat. Biotechnol. 31, 691–693 (2013). 23929340 10.1038/nbt.2655

[b21] Jiang W. Z. . Demonstration of CRISPR/Cas9/sgRNA-mediated targeted gene modification in Arabidopsis, tobacco, sorghum and rice. Nucleic. Acids Res. 41, e188 (2013). 23999092 10.1093/nar/gkt780 PMC3814374

[b22] Miao J. . Targeted mutagenesis in rice using CRISPR-Cas system. Cell Res. 23, 1233–1236 (2013). 23999856 10.1038/cr.2013.123 PMC3790239

[b23] Shan Q. . Targeted genome modification of crop plants using a CRISPR-Cas system. Nat. Biotechnol. 31, 686–688 (2013). 23929338 10.1038/nbt.2650

[b24] Jacobs T. B., LaFayette P. R., Schmitz R. J. & Parrott W. A. Targeted genome modifications in soybean with CRISPR/Cas9. BMC Biotechnol. 15, 16 (2015). 25879861 10.1186/s12896-015-0131-2 PMC4365529

[b25] Brooks C., Nekrasov V., Lippman Z. B. & VanEck J. Efficient gene editing in tomato in the first generation using the clustered regularly interspaced short palindromic repeats/CRISPR-associated 9 system. Plant Physiol. 166, 1292–1297 (2014). 25225186 10.1104/pp.114.247577 PMC4226363

[b26] Pan C. . CRISPR/Cas9-mediated efficient and heritable targeted mutagenesis in tomato plants in the first and later generations. Sci. Rep. 6, 24765 (2016). 27097775 10.1038/srep24765 PMC4838866

[b27] Feng Z. . Multigeneration analysis reveals the inheritance, specificity, and patterns of CRISPR/Cas-induced gene modifications in Arabidopsis. Proc. Natl. Acad. Sci. USA 111, 4632–4637 (2014). 24550464 10.1073/pnas.1400822111 PMC3970504

[b28] Zhang H. . The CRISPR/Cas9 system produces specific and homozygous targeted gene editing in rice in one generation. Plant Biotechnol. J 12, 797–807 (2014). 24854982 10.1111/pbi.12200

[b29] Shan Q. W., Wang Y. P., Li J. & Gao C. Genome editing in rice and wheat using the CRISPR/Cas system. Nat. Protoc. 9, 2395–2410 (2014). 25232936 10.1038/nprot.2014.157

[b30] Fan D. . Efficient CRISPR/Cas9-mediated targeted mutagenesis in Populus in the first generation. Sci. Rep. 5, 12217 (2015). 26193631 10.1038/srep12217 PMC4507398

[b31] Jia H. & Wang N. Targeted genome editing of sweet orange using Cas9/sgRNA. PLOS One 9, e93806 (2014). 24710347 10.1371/journal.pone.0093806 PMC3977896

[b32] Jaillon O. . The grapevine genome sequence suggest ancestral hexaploidization in major angiosperm phyla. Nature 449, 463–465 (2007). 17721507 10.1038/nature06148

[b33] Ferreira A. A transgenic perspective on plant functional genomics. Transgenic Res. 9, 245–260 (2000). 11131004 10.1023/a:1008967916498

[b34] Vidal J. R. . Use of gene transfer technology for functional studies in grapevine. Aust. J. Grape Wine Res. 16, 138–151 (2010).

[b35] Mullins M. G., Archie Tang F. C. & Facciotti D. Agrobacterium-mediated genetic transformation of grapevine: transgenic plants of Vitis rupestris Scheele and buds of Vitis vinifera L. Biotechnology 8, 1041–1045 (1990).

[b36] Harst M., Bornhoff B-A., Zyprian E. & Topfer R. Influence of culture technique and genotype on efficiency of Agrobacterium-mediated transformation of somatic embryos (Vitis vinifera) and their conversion to transgenic plants. Vitis 29, 99–102 (2000).

[b37] Torregrosa L., Iocco P. & Thomas M. R. Influence of Agrobacterium strain, culture medium, and cultivars on the transformation efficiency of Vitis vinifera L. Am. J. Enol. Vitic. 53, 183–190 (2002).

[b38] Ma X. . A robust CRISPR/Cas9 system for convenient, high-efficient multiplex genome editing in monocot and dicot plants. Mol. Plant 8, 1274–1284 (2015). 25917172 10.1016/j.molp.2015.04.007

[b39] Dai L. . Establishment of a picloram-induced somatic embryogenesis system in Vitis vinifera cv. chardonnay and genetic transformation of a stilbene synthase gene from wild-growing Vitis species. Plant Cell Tiss. Organ Cult. 121, 397–412 (2015).

[b40] Debolt S., Cook D. R. & Ford C. M. L-tartaric acid synthesis from vitamin C in higher plants. Proc. Natl. Acad. Sci. USA 103, 5608–5613 (2006). 16567629 10.1073/pnas.0510864103 PMC1459401

[b41] Ma X., Chen L., Zhu Q. & Liu Y. Rapid decoding of sequence-specific nuclease-induced heterozygous and biallelic mutations by direct sequencing of PCR products. Mol. Plant 8, 1285–1287 (2015). 25747846 10.1016/j.molp.2015.02.012

[b42] Iocco P., Franks T. & Thomas M. R. Genetic transformation of major wine grape cultivars of Vitis vinifera L. Transgenic Res. 10, 105–112 (2001). 11305357 10.1023/a:1008989610340

[b43] Doench J. G. . Rational design of highly active sgRNAs for CRISPR-Cas9-mediated gene inactivation. Nat. Biotechnol. 32, 1262–1267 (2014). 25184501 10.1038/nbt.3026 PMC4262738

[b44] Tsai S. Q. . GUIDE-seq enables genome-wide profiling of off-target cleavage by CRISPR-Cas nucleases. Nat. Biotechnol. 33, 187–197 (2015). 25513782 10.1038/nbt.3117 PMC4320685

[b45] Osakabe Y. . Optimization of CRISPR/Cas9 genome editing to modify abiotic stress responses in plants. Sci. Rep. 6, 26685 (2016). 27226176 10.1038/srep26685 PMC4880914

[b46] Pattanayak V. . High-throughput profiling of off-target DNA cleavage reveals RNA-programmed Cas9 nuclease specificity. Nat. Biotechnol. 31, 839–843 (2013). 23934178 10.1038/nbt.2673 PMC3782611

[b47] Fu Y. . High-frequency off-target mutagenesis induced by CRISPR-Cas nucleases in human cells. Nat. Biotechnol. 31, 822–826 (2013). 23792628 10.1038/nbt.2623 PMC3773023

[b48] Sun X. . Targeted mutagenesis in soybean using the CRISPR-Cas9 system. Sci. Rep. 5, 10342 (2015). 26022141 10.1038/srep10342 PMC4448504

[b49] Xu R. . Generation of inheritable and “transgene clean” targeted genome-modified rice in later generations using the CRISPR/Cas9 System. Sci. Rep. 5, 11491 (2015). 26089199 10.1038/srep11491 PMC5155577

[b50] Kuscu C., Arslan S., Singh R., Thorpe J. & Adli M. Genome-wide analysis reveals characteristics of off-target sites bound by the Cas9 endonuclease. Nat. Biotechnol. 32, 677–683 (2014). 24837660 10.1038/nbt.2916

[b51] Xie K., Zhang J. & Yang Y. Genome-wide prediction of highly specific guide RNA spacers for CRISPR-Cas9-mediated genome editing in model plants and major crops. Mol. Plant 7, 923–926 (2014). 24482433 10.1093/mp/ssu009

[b52] Fauser F., Schiml S. & Puchta H. Both CRISPR/Cas-based nucleases and nickases can be used efficiently for genome engineering in Arabidopsis thaliana. Plant J 79, 348–359 (2014). 24836556 10.1111/tpj.12554

[b53] Mei Y., Wang Y., Chen H., Sun Z. S. & Ju X. D. Recent Progress in CRISPR/Cas9 Technology. J. Genet. Genomics 43, 63–75 (2016). 26924689 10.1016/j.jgg.2016.01.001

[b54] Zetsche B. . Cpf1 is a single RNA-guided endonuclease of a class 2 CRISPR-Cas system. Cell 163, 759–771 (2015). 26422227 10.1016/j.cell.2015.09.038 PMC4638220

[b55] Ran F. A. . Double nicking by RNA-guided CRISPR Cas9 for enhanced genome editing specificity. Cell 154, 1380–1389 (2013). 23992846 10.1016/j.cell.2013.08.021 PMC3856256

[b56] Wang Y. . Identification of genomic sites for CRISPR/Cas9-based genome editing in the Vitis vinifera genome. BMC Plant Biol. 16, 96 (2016). 27098585 10.1186/s12870-016-0787-3 PMC4839089

[b57] Lei Y. . CRISPR-P: A web tool for synthetic single-guide RNA design of CRISPR-system in plants. Mol. Plant 7, 1494–1496 (2014). 24719468 10.1093/mp/ssu044

[b58] Livak K. J. & Schmittgen T. D. Analysis of relative gene expression data using real-time quantitative PCR and the 2(-Delta Delta C(T)) Method. Methods 25, 402–408 (2001). 11846609 10.1006/meth.2001.1262

