# Supplementary material for: CRISPR/Cas9-mediated efficient targeted mutagenesis in Chardonnay (Vitis vinifera L.)
Source: Sci Rep. 2016 Aug 31;6:32289. doi: 10.1038/srep32289 (PMC5006071; doi:10.1038/srep32289)
Supplement: Supplementary Information [file srep32289-s1.pdf]

CRISPR/Cas9-mediated efficient targeted mutagenesis in Chardonnay (*Vitis vinifera* L.)

Chong Ren[1,2], Xianju Liu[1,2], Zhan Zhang[1,2], Yi Wang[1,2], Wei Duan[1], Shaohua Li[1], Zhenchang Liang[1*]

Supplementary Information

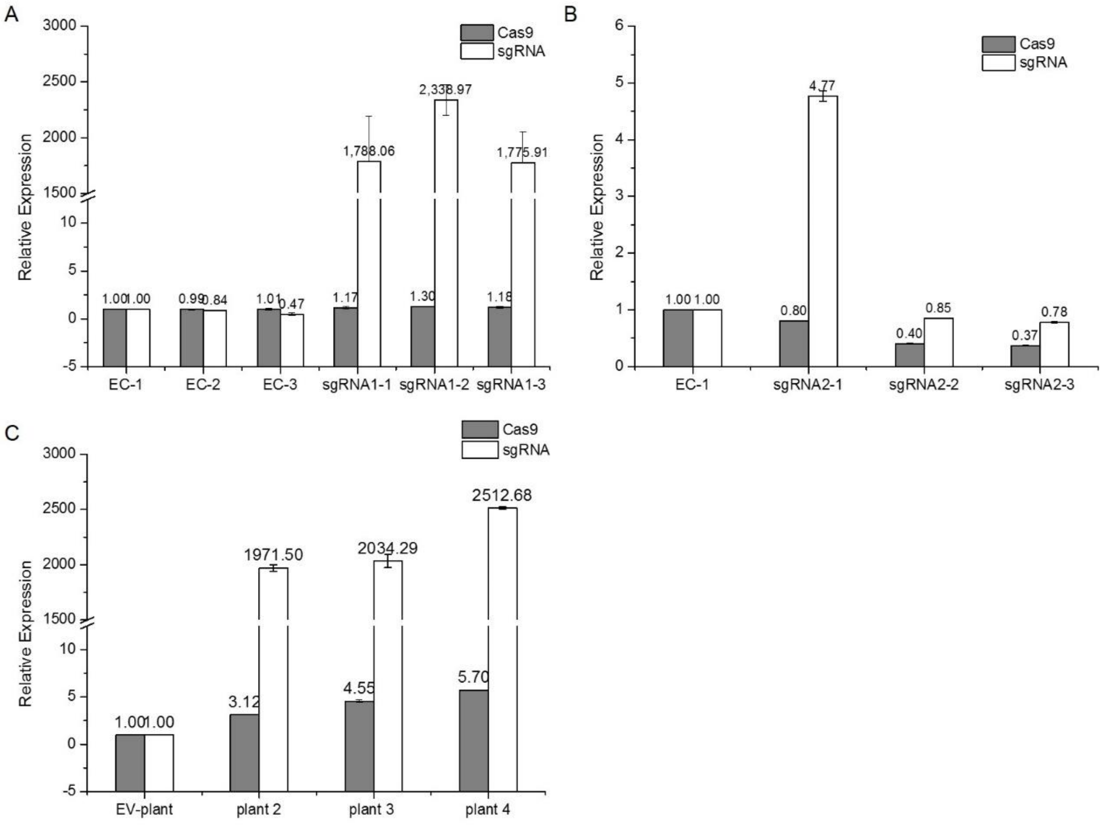

Supplementary Figure S1. Expression analysis of *Cas9* and sgRNAs by qRT-PCR. Expression levels of *Cas9* and sgRNA in sgRNA1-CMs (A), sgRNA2-CMs (B) and sgRNA1-plants (C) were measured, respectively. The EV-CM (EC) or EV-plant with no detected mutation was used as control. The grape *Actin 1* was used as the internal control.

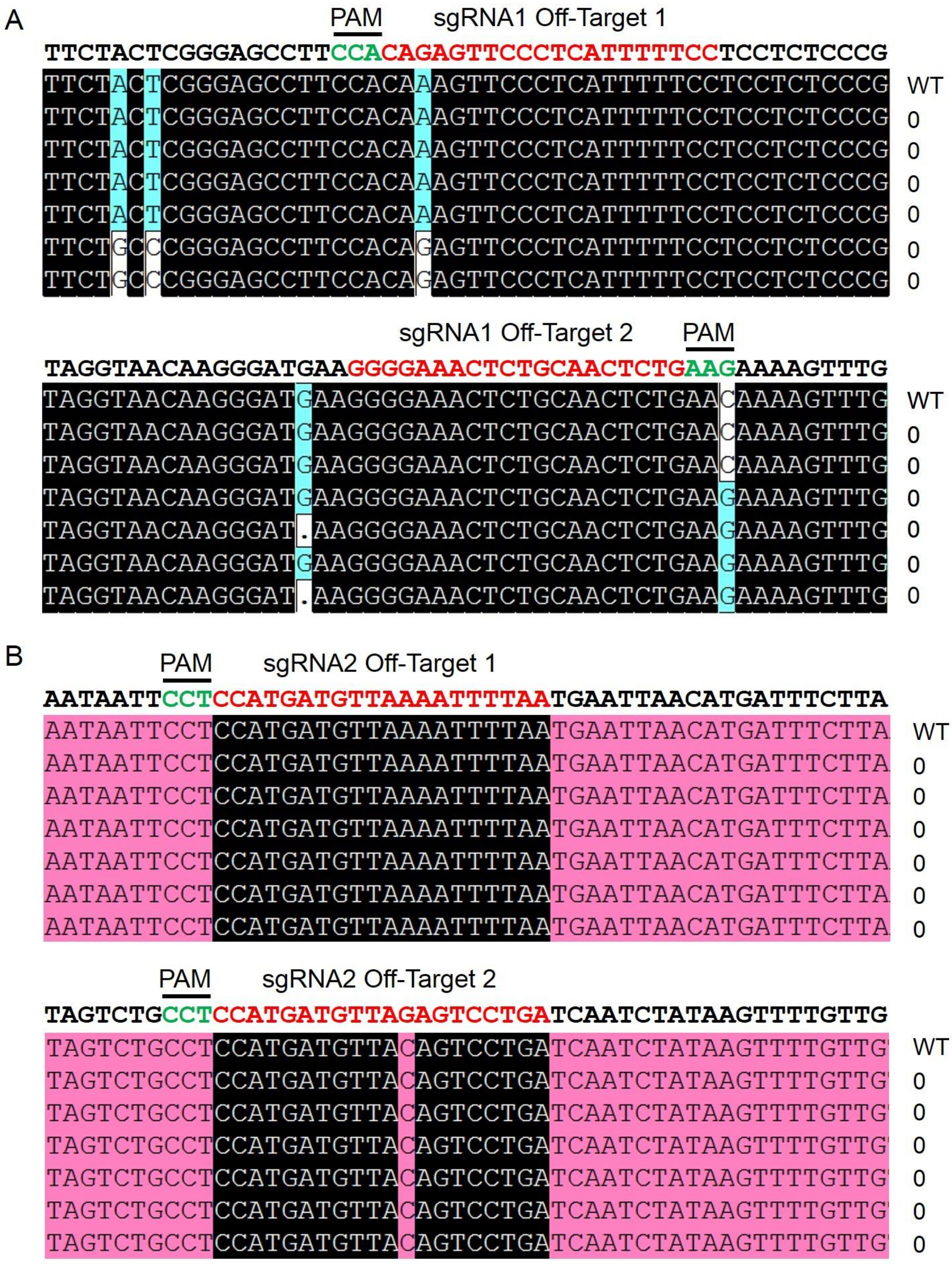

Supplementary Figure S2. Analysis of off-target events in putative off-target sites. (A) The sequencing results of DNA fragments containing the putative off-target sites of sgRNA1. (B) The sequencing results of DNA fragments containing the putative off-target sites of sgRNA2.

Supplementary Table S1. The sequences and GC contents of sgRNAs.

Target gene	sgRNA	Sequence	Target site	sgRNA GC content
IdnDH	1	GGGGAAAGGAGGCAACTCTG	exon 1	60.0%
	2	TCCTAACTTTAACATCATGG	exon 2	35.0%

Supplementary Table S2. Primers used in this study.

Primer name	Primer sequence (5'→3')	Aims
IdnDH-F	AATACTTGGAGCCCCATCCAAT	Amplification of *IdnDH*
IdnDH-R	GTGAGAGTTGCGGAGATGACT	
sgRNA1-off1-F	AACCTGATGGCCGTTGTTCG	Off-target analysis
sgRNA1-off1-R	GACAGACGTTCAGCCACCAG	
sgRNA1-off2-F	CCTTGTGGACCCTCACCACT	
sgRNA1-off2-R	GGGCAGAATCTGTGCTCGC	
sgRNA2-off1-F	ATTCCACAAGCACAAAGTCTTCA	
sgRNA2-off1-R	CTATAAGGCACAAAGGGTCCCA	
sgRNA2-off2-F	CCCATCAACTTACAAGTTACAACA	
sgRNA2-off2-R	CTTCCAAGAGAGCTTTGACGA	
Actin1-F	CAGCAGATGTGGATCTCAAA	qRT-PCR
Actin1-R	CTGTGGACAATGGAAGGAC	
sgRNA1-F	GGGAAAGGAGGCAACTCTGG	
sgRNA1-R	CCGACTCGGTGCCACTTT	
sgRNA2-F	GGAGTTTTAGAGCTAGAAATAGCAA	
sgRNA2-R	CTCGGTGCCACTTTTTCAAG	
Cas9-F	CAGATTCGCCTGGATGACCA	
Cas9-R	ATCCGCTCGATGAAGCTCTG	
Hyg-F	GTCCGTCAGGACATTGTTGGAGCC	Identification of T-DNA
Hyg-R	GTCTCCGACCTGATGCAGCTCTCGG	
